# Attribution of original contribution in large datasets in the era of multi‐omic studies

**DOI:** 10.1002/acn3.51571

**Published:** 2022-06-02

**Authors:** Payam Mohassel, Eleonora Guadagnin, Carsten G. Bönnemann

**Affiliations:** ^1^ Neuromuscular and Neurogenetic Disorders of Childhood Section National Institutes of Neurological Disorders and Stroke NIH Bethesda Maryland USA; ^2^ Present address: Moderna Inc Cambridge Massachusetts 02139 USA

Dear Dr. Kessler,

We are very appreciative of Dr. Jiménez‐Mallebrera's supportive assessment^1^ of our manuscript on transcriptomic analysis of collagen VI‐related dystrophy (COL6‐RD) muscle biopsies in their letter to the editor in this issue. We were also encouraged with the considerable overlap of findings between our study published earlier this year^2^ and their pioneering and insightful microarray analysis of six COL6‐RD muscle biopsies published in 2013.^3^

It is relevant to highlight that our study was not designed as a comparative study to previous datasets. Instead, it was designed to identify molecular signatures that enhance in correlation with pathologic disease severity in COL6‐RD muscle biopsies. In addition to collecting a larger sample size (*n* = 20 for microarray and *n* = 22 for RNA‐Seq), grouping of biopsies based on histologic severity prior to transcriptomic analysis and bioinformatic tools such as polyserial correlation analysis were thus used to extend upon similar prior studies. Accordingly, the main finding of our study, upregulation of TGFβ‐related pathway as a master regulator, had not been highlighted by previous studies even though some of the related genes were listed to be dysregulated in other studies as well.

Dr. Jiménez‐Mallebrera also raised the specific issue about the novelty of two genes highlighted in our manuscript, *CILP* and *MGP*. In our combined microarray/RNA‐Seq dataset, *CILP* and *MGP* rank 10th and 24th among 461 upregulated genes, respectively. We of course acknowledge that the genes are also listed in their dataset, where *CILP* and *MGP* rank 269th and 219th in a list of 655 upregulated genes. However, *MGP* is listed as part of extracellular matrix‐related genes, validated by qPCR, but is not discussed further, and *CILP* is neither highlighted nor discussed in the paper (it is included in supplemental table S4).^3^ Importantly in this context, in our polyserial correlation analysis both *CILP* and *MGP* were among the list of top 20 dysregulated genes with highest correlation scores (out of 412), suggesting their upregulation correlated with histologic severity (table S5).^2^ Thus, the conspicuous upregulation of these genes across different methods of data analysis prompted us to validate the findings by qPCR and to speculate about their function and connection with the TGFβ pathway and muscle transdifferentiation in our discussion.

With generation of large datasets in the ‐omics age, it is only natural and in fact a part of the discovery process that high quality or difficult to obtain datasets will be re‐done or re‐analyzed by different scientists with different or evolving analytic and interpretative methods, which can in fact lead to novel insights and discoveries. While it is in fact reassuring that there is no fundamental disagreement between our datasets, we respectfully maintain that our recognition of the special behavior of these specific genes in our dataset, their validation and discussion in the context of COL6‐RD, and their connection with the TGFβ pathway in our dataset constitutes an original contribution. We have also now made our dataset available to the public ahead of schedule and we hope it will also form the basis for re‐analysis and interpretation or meta‐analysis as suggested by Dr. Jiménez‐Mallebrera, perhaps resulting in novel insights not recognized by us. If so, it would again justly constitute a novel discovery on which we would lay no claim.

There has been incredible progress in the generation of large ‐omics datasets over the last decade, providing powerful tools to study disease mechanisms. Although significant advances in minimum information standards for single‐omics datasets have been made, methods in data generation and analysis vary widely. Large‐scale programs such as the NIH Big Data to Knowledge (BD2K) initiative^4^ or commercial data analysis platforms (e.g., Qiagen Ingenuity Pathway Analysis) among others aim to address some of these limitations. With this, we are hopeful that ours and similar studies will be continued to be analyzed to provide mechanistic insights and help facilitate the development of therapeutic targets and biomarkers for congenital muscular dystrophies including COL6‐RD, which is the primary goal of all of us.

## Conflict of Interest

The authors report no conflict of interest.
